# Targeting the Tumor Microenvironment: The Protumor Effects of IL-17 Related to Cancer Type

**DOI:** 10.3390/ijms17091433

**Published:** 2016-08-30

**Authors:** Joseph Fabre, Jerome Giustiniani, Christian Garbar, Frank Antonicelli, Yacine Merrouche, Armand Bensussan, Martine Bagot, Reem al-Dacak

**Affiliations:** 1 Institut Jean Godinot, Unicancer, 1 rue du Général Koenig, F-51726 Reims, France; jerome.giustiniani@gmail.com (J.G.); Christian.GARBAR@reims.unicancer.fr (C.G.); yacine.merrouche@reims.unicancer.fr (Y.M.); 2 Université Reims-Champagne-Ardenne, DERM-I-C, EA7319, 51 rue Cognacq-Jay, F-51095 Reims, France; frank.antonicelli@univ-reims.fr; 3 Centre Hospitalo-Universitaire Henri Mondor, Service de Radiothérapie, 51 Avenue du Maréchal de Lattre de Tassigny, F-94010 Créteil, France; 4 Institut National de la Santé et de la Recherche Médicale (INSERM) U976, Hôpital Saint Louis, F-75010 Paris, France; martine.bagot@aphp.fr; 5 Université Paris Diderot, Sorbonne Paris Cité, Laboratoire Immunologie Dermatologie & Oncologie, UMR-S 976, F-75475 Paris, France; reem.al-daccak@inserm.fr; 6 OREGA Biotech, 69130 Ecully, France

**Keywords:** interleukin 17 (IL-17), cancer, tumor microenvironment, immunotherapy

## Abstract

The inflammatory process contributes to immune tolerance as well as to tumor progression and metastasis. By releasing extracellular signals, cancerous cells constantly shape their surrounding microenvironment through their interactions with infiltrating immune cells, stromal cells and components of extracellular matrix. Recently, the pro-inflammatory interleukin 17 (IL-17)-producing T helper lymphocytes, the Th17 cells, and the IL-17/IL-17 receptor (IL-17R) axis gained special attention. The IL-17 family comprises at least six members, IL-17A, IL-17B, IL-17C, IL-17D, IL-17E (also called IL-25), and IL-17F. Secreted as disulfide-linked homo- or heterodimers, the IL-17 bind to the IL-17R, a type I cell surface receptor, of which there are five variants, IL-17RA to IL-17RE. This review focuses on the current advances identifying the promoting role of IL-17 in carcinogenesis, tumor metastasis and resistance to chemotherapy of diverse solid cancers. While underscoring the IL-17/IL-17R axis as promising immunotherapeutic target in the context of cancer managing, this knowledge calls upon further in vitro and in vivo studies that would allow the development and implementation of novel strategies to combat tumors.

## 1. Introduction

Tumor cells have enhanced capacities of proliferation, neo-angiogenesis development and distance seeding under the form of metastases [1,2]. The tumor microenvironment (TME), which comprises malignant and non-malignant cells distinguished by specific markers and interacting in a dynamic fashion, is an important aspect of cancer biology that contributes to tumor initiation, tumor progression and responses to therapy [3,4,5]. Cells and molecules of the immune system are a fundamental component of the TME. Although critical for anti-tumor responses, cells of the immune system including macrophages, neutrophils, mast cells, dendritic cells (DCs) and lymphocytes can also promote the development and progression of almost every solid tumor [6,7,8]. Tumor cells counterattack the host’s immune cells detouring them to their own profit and evading elimination [9]. They often secrete a variety of cytokines and mediators creating a self-entertaining inflammation of the TME that is favorable to tumor development and progression [10].

Recently, a subset of T helper (Th) lymphocytes secreting mainly the pro-inflammatory IL-17 cytokines, the Th17 cells, has gained considerable attention, given their contribution to infectious, auto-, and cancer immunity [11]. Consequently, the IL-17 pro-inflammatory cytokines have become a key therapeutic target in a variety of chronic inflammatory diseases. Because inflammation is also tightly correlated to cancer development [12], these cytokines have been also intensively investigated in the context of cancer development and progression. Recent research provided substantial insights into the mode of action of Th17 and IL-17 cytokines in a variety of tumors. Lessons are learned and paradigms are changing: IL-17 cytokines are double-edged agents acting in a cancer-type depending manner as anti- and protumor cytokines. If respectively targeted, the IL-17/IL-17R axis could be part of the dynamic and durable mechanisms that might promote tumor regression. We discuss the hurdles, lessons, and advances accomplished in the field through the progressive journey of IL-17 family toward tumor immunotherapy.

## 2. The IL-17/IL-17R Axis

### 2.1. Tumor Infiltrating Lymphocytes and Th 17 Cells

Tumor infiltrating lymphocytes present a minor population of healthy and cancer patients’ pool of peripheral and lymph nodes T lymphocytes but are found at a high concentration in the microenvironment of diverse types of cancers [13,14,15]. The intensity of TIL infiltration to tumors often correlates with the stage of the disease [16]. TIL comprise various subsets of T lymphocytes, among which is the subset of Th17 lymphocytes.

Th17 cells have been extensively studied over the last five years. They are an independent lineage of Th lymphocytes and are characterized by a specific cytokine secretion profile, transcription regulation and immune functions [17]. Th17 play important role in infection since they repel against diverse microbes and are key mediators of inflammation in a variety of inflammatory and autoimmune disorders including psoriasis, rheumatoid arthritis and inflammatory bowel diseases [18]. The development of Th17 lineage is controlled by RORγt, STAT3 and IFN regulatory factor-4 transcription factors and necessitates the exposure to a variety of cytokines [19]. In mouse, lymphocyte engagement in the Th17 pathway needs the exposure to TGF-β plus IL-6 or IL-21 [20] as well as IL-23 [21]. In human, IL-1 is the cornerstone of human Th17 cells differentiation, and can be potentiated by a combination of IL-23, IL-6 and TGF-β [22,23]. Besides cytokines, the activation of antigen-presenting cells, the DCs, through the Toll-like receptor (TLR) and bacterial sensor nod2 programs them to polarize human memory T cells towards the Th17 lineage [24].

Similar to other T lymphocytes subsets, Th17 cells also infiltrate cancers. Within the tumor microenvironment, the infiltrating Th17 cells are often abundant at a proximity to the tumor mass. Phenotypically, these cells, to which we will refer to as TIL-Th17 cells, express memory-like markers (CD45RA−CD45RO+), CD49 integrins, and surface receptors allowing their traffic to peripheral tissues including CXCR4, CCR6 and C-type lectin CD161 [25,26]. However, TIL-Th17 cells do not express CCR2, CCR5 and CCR7, which limit their capacity to access the lymph nodes [27]. This configuration may be responsible for Th17 lymphocytes stagnation in the tumor microenvironment where the levels of CCL20 and CXCL12 are high [28]. CCL20 can be also produced by Th17 [29], which could self-consolidate their adhesion to the tumor site.

Compared to conventional effector T cells, Th17 phenotype comprised low levels of granzyme B and activation markers HLA-DR and CD25. This observation is in favor of an impossibility to initiate cytotoxic killing. Besides, Th17 express minimal PD-1 and forkhead box P3 (FOXP3), which make them distinct from immune-suppressive regulator T cells (Treg) [30].

### 2.2. IL-17 and IL-17 Receptor Family

Early studies with rodent models described in T cell hybridoma a cDNA sequence coding for a mRNA sharing characteristics with cytokines [31]. Primarily designated as CTLA8, it was termed interleukin-17 (IL-17) after its cloning from a cDNA library [11]. Today, the IL-17 is a family of pro-inflammatory cytokines implicated in a variety of immune responses and is composed of six members, from IL-17A to IL-17F.

IL-17A and -F share 50% homology and are the closest members [32]. They are secreted as IL-17A and IL-17F homodimers and also as IL-17A/F heterodimers [33]. In term of activity, IL-17F is less potent than IL-17A, and the heterodimer has an intermediate efficacy [34]. The functions of IL-17B, IL-17C and IL-17E are less defined. Nonetheless, IL-17E (also referred to as IL-25), which shares the lowest homology with IL-17A, was involved in allergy reactions and in immunity against parasites [35]. Although their production is the hallmark of Th17 cells, both IL-17A and -F can also be produced by γδT cells, natural killer T (NKT) cells, neutrophils and eosinophils [36].

**Figure 1:**
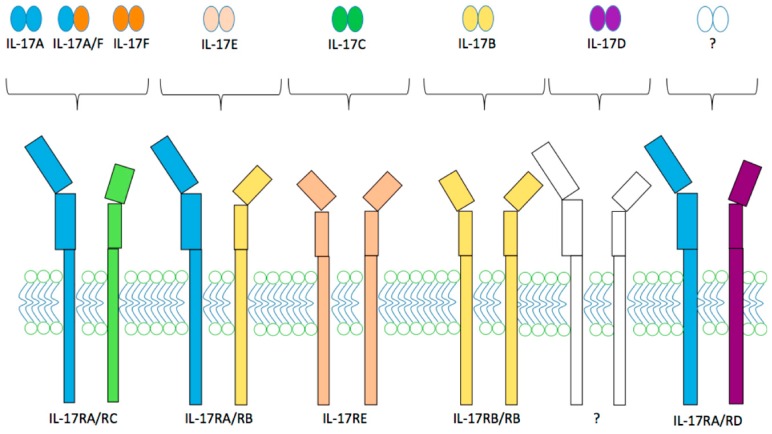
Interleukin 17 and interleukin 17 receptor family members.

The receptor of IL-17 (IL-17R) is a transmembrane protein composed of a 27 amino acid (aa) N-terminal signal peptide, a 293 aa extracellular domain, a 21 aa transmembrane domain and a cytoplasmic tail of 525 aa [37]. Particular motives have been identified within these domains: fibronectin type III (FnIII) regions in the extracellular portion of the protein and similar expression to fibroblast growth factor genes (SEFIR) motif inside the cytoplasmic tail. Five members of the IL-17R family have been identified so far and designated as IL-17RA, IL-17RB, IL-17RC, IL-17RD and IL-17RE (Figure 1). Each of these members is a subunit that needs to associate with another one to form the functional receptor [38]. The subunit IL-17RA is ubiquitous, and is encoded by a gene situated on chromosome 22, while others are encoded by a cluster on chromosome 3 [23,33]. It is also a common co-receptor subunit for other members of the IL-17 family. Conversely, IL-17RC subunit was an obligate co-receptor for IL-17RA to mediate IL-17A, IL-17F and IL-17A/F signaling [39]. Other members of the IL-17 family, IL-17B, IL-17C, and IL-17E respectively bind IL-17RB, IL-17RA/RC, and IL-17RA/RB [40]. IL-17D as well as IL-17RD matches remain unfound yet [36]. In any case, ligand fixation activates IL-17RA and transduces signal through the phosphorylation of Mitogen Activated Protein Kinases (MAPK) and Nuclear Factor-κB (NF-κB) via TNF Receptor Associated Factor-6 (TRAF6). Interaction with NF-κB protein (Act1) has also been reported [41]. To comfort this, Act1 knockdown experiences showed abrogation of IL-17 induced inflammatory gene expression and NF-κB activation [42]. The structural data about SEFIR domains of IL-17RA, IL-17RB and IL-17RC are accumulating and suggest a key role of the αC-helix in the SEFIR-SEFIR interactions with Act1 [43]. Secondary to Act1 and TRAF6 activation, Iκβ kinase (IKK) phosphorylates p105, which releases Tumor Progression locus 2 (TPL2). TPL2 then phosphorylates MEK1, which activates ERK1 and ERK2 and ultimately leads to transcription factors phosphorylation and gene expression modulation [44].

### 2.3. IL-17 and Cancer

If the efficacy of IL-17 pathway inhibiting therapies in inflammatory disease like rheumatoid arthritis or psoriasis has been clearly established [45], it still has to be validated as a target for cancer treatment given its double-edged role in cancer. Indeed, since its primary detection in human cancers including breast, gastric and prostate cancer [46,47,48,49,50,51], the role of IL-17 in oncology has been highly debated and controversial [52].

#### 2.3.1. IL-17 and Cervical Cancer

One of the earliest publications arguing in favor of a deleterious tumor enhancing effect of IL-17 was the study from Tartour et al. [53]. They observed that cervical cancer cell cultured with IL-17 had an increased production of both IL-6 and IL-8 mRNA and proteins levels. Although no direct effect on proliferation had occurred in vitro, the tumor size was increased when nude mice were transplanted with two cell lines transfected with IL-17-encoding-cDNA as compared to the parent tumor. Of note, IL-6 level and macrophage number was raised at the tumor site. Punt et al. also reported cell index-stimulating effects of IL-17 and observed it was present mainly in neutrophils (66%), mast cells (23%) and at a lesser level in innate lymphoid cells (8%). From patients’ tissue samples, they observed that a higher number of neutrophils was correlated with a poorer survival [54].

#### 2.3.2. IL-17 and Breast Cancer

Several articles brought evidence for a protumor role of IL-17. Lyon et al. confronted the blood levels of diverse cytokines from 35 patients newly diagnosed with breast cancer (BC) to patients with negative breast biopsy [55]. They observed that IL-17, IL-6 and GCS-F were significantly more elevated in breast cancer patients than in control though no correlation with prognosis was made. Zhu et al. were the first to propose a study to analyze the expression in situ of IL-17 in BC. On immunostaining, IL-17 was located particularly to the peritumoural area concomitantly to a macrophage infiltration. Then, they evaluated Four BC cell lines (MDA-MB231, MDA-MB435, MCF7 and T47D) on matrigel invasion assay. IL-17, as well as TNF, markedly increased invasion for MDA-MB231 and MDA-MB435. When adding matrix metalloproteinase (MMP) inhibitors to cell cultures, the IL-17-dependent invasion was inhibited [56]. Other authors suggested that tumor favoring effects of IL-17 may occur via an increase in suppressive functions of myeloid-derived suppressor cells (MDSCs) through the CXCL1/5–CXCR2 axis. In this study, anti IL-17-blocking antibodies were reported to dramatically decrease tumor growth and the number of MDSCs in mice [13]. Angiogenesis-promoting effects of IL-17 in breast tumor cell grafts in vivo as measured by microvascular density has also been reported [57]. In contrast with these results, some authors reported anti-tumor activity of the IL-17 family members. Furuta et al. observed caspase-dependent pro-apoptosis effects of IL-17E on culture cells as well as xenografts [58].

Nevertheless, our team also observed that human breast cancer cell lines expressed IL-17RA and IL-17RC, and stimulation with IL-17A recruited the MAPK pathway by upregulating phosphorylated ERK1/2 as reported in previous studies [59]. This mechanism lead to enhanced migration, invasion and resistance to chemotherapy, and was abrogated by anti-IL17A antibodies [60].

Our next step was to further assess the molecular signaling after stimulation of human breast cancer cell lines with IL-17A and IL-17E. The result was an induction by both cytokines of the phosphorylation of c-RAF, ERK1/2 and p70 S6 Kinase, which are known to be involved in the proliferation and survival of tumor cells [61]. Besides, unlike findings by other authors [58,62,63], IL-17A or IL-17E did not induce apoptosis in IL-17RB-expressing human breast cancer cells but conversely exacerbated cell resistance to docetaxel [61]. Another argument in favor of a protumor impact of IL-17A and -E was the detection of an enhanced generation of low molecular weight forms of cyclin E (LMWE) in the four different cell lines (MCF7, T47D, MCF10A and IJG-1731) [61], which has been described as a negative factor in cancer [64].

#### 2.3.3. IL-17 and Prostate Cancer

Chronic inflammation and eventually atrophy are implicated in prostate cancer [65]. The first report of the presence of an IL-17R-like receptor in prostate cells was in 2002 [46]. The year after, another study confirmed that IL-17 expression was low in normal prostate cells, whereas it was elevated in 58% of cancer cells and 79% in benign prostatic hypertrophy cells. Concerning IL-17 receptor, it was ubiquitously detected [47]. Only years later, You et al. identified the reported transmembrane protein to be IL-17-RC of which they reported several isoforms both in hormone-dependent and independent prostate cancers [66]. Moreover, a study analyzed cell signaling mediated by IL-17A ex vivo, and found that ERK pathway and NF-κB were activated but cell growth was not modified. Besides, several chemokines (CXCL1, CXCL2, CCL2, and CCL5) and IL-6 expression level were increased [67].

Liu et al. investigated whether obesity played a role in prostate cancer (PC) development [68]. In a mice model of obesity they found a higher rate of PC [69]. Obese subjects developed a chronic inflammatory state with increased serum levels of IL-17, insulin, and insulin-like growth factor 1 (IGF1). They also observed that hyperinsulinemia reinforced IL-17-induced expression of downstream proinflammatory genes with increased levels of IL-17RA, resulting in the development of more invasive prostate cancer. Glycogen synthase kinase 3 (GSK3) is constitutively attached to the phosphorylation of IL-17RA at T780 and its activation leads to ubiquitination and proteasome-mediated destruction of IL-17RA, which ultimately blocks IL-17-mediated inflammation [68]. Concordantly, phosphorylated IL-17RA was decreased, and its IL-17RA mRNA levels were raised in the proliferative human prostate cancer cells compared to the normal cells. Besides, Insulin and IGF1 enhanced IL-17-induced inflammatory responses through suppressing GSK3 in cultured cell lines and in obese mouse models of prostate cancer indicating crosslink between the insulin receptor pathway and IL-17 signaling [68]. From a therapeutic point of view, GSK3-targeting may lead to the suppression of IL-17-mediated inflammation and could prevent appearance of prostate cancer in obese men. The same authors very recently added evidences from murine experiences that IL-17-mediated prostate cancer promotion may occur through epithelial to mesenchymal transition via MMP7 enhancement [70]. This mechanism is concordant with a progressive transformation of prostate cancer cells exposed to chronic inflammation via IL-17-recruiting of inflammatory cells and ultimately a loss of adhesion. The improved invasiveness through metalloproteinase expression allows them to digest extracellular matrix as well as the basal membrane and thus expand.

#### 2.3.4. IL-17 and Gastric Cancer

Only a few studies evaluated IL-17 in gastric cancer. Zhou et al. investigated the immortalized AGS human gastric adenocarcinoma cell line. They demonstrated that IL-17A and possibly IL-17F could initiate transduction pathways, increase expression of MAPKs and recruit neutrophils participation in gastric inflammation and thus promote cancer progression through IL-17R docking. These effects were significantly abrogated by disrupting IL-17RA or IL-17RC signaling, therefore evocating their participation in gastric cancer [71]. Interleukin-17B acts in a paracrine fashion since it has also been detected locally in gastric cancer but only at low levels, whereas the expression of its receptor IL-17RB was increased and correlated with poor prognosis. Surprisingly, IL-17E expression was absent in the stomach. Evaluation of the IL-17B/17-RB axis in gastric cancer cells, reported that it promoted proliferation and migration as well as stemness phenotype as assessed by the detection of stem cells markers Oct4, Nanog, Lgr5, Sall4 [72]. The mechanism was an activation of the AKT/GSK-3β/β-catenin pathway.

#### 2.3.5. IL-17 and Colon Cancer

Colorectal cancers develop from normal colonic epithelium in a four-step progression of gene alterations [73]. The study by Cui et al. evaluated the dynamic variations of the expression of IL-17A in the tumor microenvironment during the colorectal adenoma-carcinoma sequence. Results are in favor of a progressive increase of IL-17 mRNA level throughout the sequence which was correlated with dysplasia severity. By immunohistochemistry (IHC), they confirmed these results by observing that Th17 presence was growing gradually in both stroma and adenomatous/cancerous epithelium. Th17-stimulating factors like IL-1β, IL-6, IL-23, TGF-β and housekeeping gene β-actin were also increased in real-time PCR, which indirectly reflects an activation of Th17 cells along the sequence [74]. To complete this observation, Al-Samadi et al. reported an increased level of IL-17B in CRC in both epithelial and stromal compartments, while IL-17F was decreased. Concerning IL-17E, no difference was noted between altered and healthy tissues [75]. For IL-17C the expression pattern was dependent on the grade of the differentiation.

In 2016, Housseau et al. reported a redundant role for adaptive and innate γδT17 cell-derived IL-17 in a model of bacteria induced colon carcinogenesis [76]. Notably, they observed that knocking out STAT3 in CD4+ T cells delayed tumorigenesis but failed to definitely suppress colonic tumors appearance. IL-17 appeared critical for the emergence of colonic tumors, although this cytokine is secreted by another source than cancer cells. The source responsible for this relay of IL-17 secretion may be mucosal γδT17 cells because genetic ablation in ETBF-colonized Th17 deficient mice prevented the late emergence of colonic tumors. When they examined human colon cancer samples, both Th17 and γδT17 cells were found. Finally, when they inoculated MC38 colon cancer cell line in IL-17^−/−^ mice, it had an enhanced growth and developed metastases to the lungs more frequently than nondeficient mice. The explanation could be a reduction in IFNγ producing NK and CD8+ cells [27].

#### 2.3.6. IL-17 and Lung Cancer

In 2015, Pan et al. investigated the impact of IL-17 in non-small cell lung cancer (NSCLC) [77,78]. Ex vivo, they observed that IL-17 could induce VEGF secretion in cancer cell lines. This effect was dependent of the STAT3-Gα–Interacting Vesicle-associated protein (GIV) pathway and was abolished when cells where exposed to small interfering RNA (siRNA) [77]. In patients, they observed that those with increased levels of serum IL-17 had a poorer survival and an enhanced angiogenesis compared to healthy control [78]. To comfort that, exposure of three different NSCLC cell lines to IL-17 has also been reported to increase neoangiogenesis and to promote in vivo tumor growth in SCID mice through a CXCR-2-dependent mechanism. IL-17 up-regulated several pro-angiogenic CXC chemokines including CXCL1, CXCL5, CXCL6 and CXCL8. Inhibition of IL-17 with monoclonal antibodies abolished this up-regulation. Noteworthy, direct stimulating effect on cell cultures was not observed [79].

#### 2.3.7. IL-17 and Skin Cancer

Several studies reported effects of IL-17 on tumor progression, growth and migration in skin cancer cell culture as well as in mice [80,81]. Both basal and squamous cell cancer cell types were responsive to IL-17 stimulation. Similar to other cell types, cytoplasmic adaptor Act1 has been reported as critical for IL-17R signaling and ultimately tumor formation [82]. The authors also described a novel IL-17-mediated cascade starting from IL-17R and leading to ERK5 activation via recruitment of Act1, TRAF4 and MEKK3. At a genetic level, the metalloreductase Steap4 and transcription factor p63 were reported to be overexpressed at the end of the cascade and to create a positive feedback through p63-mediated TRAF4 expression.

Wang et al. studied the role of IL-17 in cell lines growth of melanoma (B16) and bladder carcinoma (MB49) [83]. Both had a reduced expansion in IL-17^−/−^ mice, and were enhanced in IFNγ^−/−^ mice as a consequence of an elevated intra-tumor IL-17 level. IFNγ probably plays a minor or an upstream role since double knockout (KO) mice are resistant to tumor cell growth like IL-17^−/−^. In vitro, the cell proliferation promoting-effect was modest. In each cell line, IL-6 production was stimulated by signal transducer and activator of transcription (Stat3). The same was observed in tumor associated stromal cells such as fibroblasts, endothelial cells and dendritic cells. The pro-angiogenic effects of IL-17 such as angiogenic factor secretion by endothelial cells and improved cell migration were also dependent of STAT3 activation. In absence of IFNγ, tumors were characterized by an important production of IL-17 and IL-6 by tumor infiltrating cells and tumor cells. When blocking IL-6, tumor progression was partially reversed indicating that the protumor activity of IL-17 needs IL-6 in a STAT3-dependent pathway.

#### 2.3.8. IL-17 and Brain Tumors

IL-17 has not been suggested to play a role in brain tumors except for gliomas. A clinical study compared blood levels of IL-17 in 80 brain tumors versus 26 healthy patients and found it was elevated in 30% of gliomas, 4% of meningioma, 5.5% of schwannoma, and none of the control group [84]. When exposed to IL-17, diverse glioma cell lines showed an increased expression of IκB-α mRNA and IκB-α protein degradation. Besides, IL-17 also stimulated IL-6 and IL-8 alone and in a potentiated fashion when combined with IL-1β [85]. In vivo, the presence of Th17 lymphocytes and IL-17A mRNA was demonstrated in glioma from mouse and human yet without assessing their impact in that situation [86]. The presence of Th17 cells in the glioma microenvironment was further investigated by Cantini et al. [87]. The authors reported Th17 and Treg cells infiltration in mouse glioma to be time-dependent. Then, they injected spleen-derived Th17 from naive (nTh17) or glioma-bearing mice (gTh17) concomitantly to GL261 glioma cell lines in immune-competent mice. All Th17 lymphocytes showed high levels of IL-17mRNA, variable levels of IL-10 and IFNγ but lacked Foxp3. Survival was significantly shorter and tumor size larger in mice injected with gTh17 than nTh17. Analysis of the microenvironment showed that in tumors co-injected with nTh17, high expression of IFNγ and TNF was present, whereas, in comparison to gTh17 IL-10 and TGF-β, were at least two times more expressed. Note that direct exposure of glioma cell lines to IL-17 did not stimulate cell growth. Hu et al. suggested indirect tumor-promoting effects of IL-17 via accelerated angiogenesis [88]. Glioma graft growth in mice transfected with an IL-17 vector was superior to the mock vector, as was the mRNA expression levels of CD31 in tumor tissues.

Some authors reported tumor-protective effects of Tregs in glioma. IL-17+ Tregs were identified in abundance in surgically removed high-grade glioma, and co-culture provoked an inhibition of CD8+ T cells proliferation. Tregs suppressive effects are probably mediated by TGF-β and IL-17 because selective antibodies against each of the cytokine blocked the inhibition of CD8+ and when both IL-17 and TGF-β were targeted, the effect was potentiated [89].

Recently, functional IL-17R has been detected in glioma stem cells and suggested as an interesting target [90].

### 2.4. IL-17 and Hepatocarcinoma

Zhang et al. observed in 2009 that IL-17+ cells were strongly represented in human hepatocellular carcinoma (HCC) and their level was correlated with both microvessel density in tissues and poor survival for patients [91]. They also reported that most of these IL-17+ cells were Th17 (CD4+) and a significant number amongst them were CD8+ T cells. Later, the same team found that tumor-activated monocytes could enhance the proliferation of these CD8+ T cells [92].

### 2.5. IL-17 and Sarcoma

The activation of IL-17R at the surface of synovial sarcoma cells by IL-17A [64] was found to recruit ERK1/2, p38 MAPK and c-Jun N-terminal kinases (JNKs), and then to activate AP-1, finally leading to an increase of MMP-3 mRNA and protein expression, which has been shown to stimulate tumor development in breast and lung [93,94]. On the other hand, an intact IL-17/IL-17R axis has been reported to improve immunogenic cell death after chemotherapy [95]. Another study by O’Sullivan et al. also regroups experiments on sarcoma cells lines among other cancer types with a focus on IL-17E [96]. They observed that the cytokine mediated tumor rejection through the recruitment of NK cells probably by stimulating the production of chemokine MCP-1.

## 3. Conclusions

Very soon after its discovery, researchers have been exploring the relationships between the IL-17 pathway and cancer development and progression. The presence of IL-17 and Th17 cells has been confirmed in almost all types of invasive cancers rare or frequent. Taken together, our data support cell proliferation, tumor growth and progression and treatment resistance through cell IL-17R signaling activation and probably crosslinks with other receptors such as EGFR, or IGFR. Analysis of patients’ blood or tissue samples was more often reported in favor of a negative impact of high values of IL-17 and IL-17-secreting cells. Nevertheless, a few studies have also reported anti-tumor activity. The presence of regulator T lymphocytes, as well as cytokines other than IL-17 present in the microenvironment modulate the immune response and orientate the balance towards cancer immunogenicity or immunosuppression. Our present work underscore IL-17/IL-17R axis as a promising immunotherapeutic target, and calls upon further in vitro and in vivo studies that would allow the development of novel strategies to combat tumors.
